# Cathepsin L plays a key role in SARS-CoV-2 infection in humans and humanized mice and is a promising target for new drug development

**DOI:** 10.1038/s41392-021-00558-8

**Published:** 2021-03-27

**Authors:** Miao-Miao Zhao, Wei-Li Yang, Fang-Yuan Yang, Li Zhang, Wei-Jin Huang, Wei Hou, Chang-Fa Fan, Rong-Hua Jin, Ying-Mei Feng, You-Chun Wang, Jin-Kui Yang

**Affiliations:** 1 Department of Endocrinology, Beijing Tongren Hospital, Capital Medical University, Beijing, China; 2 Division of HIV/AIDS and Sex-Transmitted Virus Vaccines, Institute for Biological Product Control, National Institutes for Food and Drug Control (NIFDC), Beijing, China; 3 Department of Science and Technology, Beijing Youan Hospital, Capital Medical University, Beijing, China; 4 Division of Animal Model Research, Institute for Laboratory Animal Resources, National Institutes for Food and Drug Control, Beijing, China

**Keywords:** Infectious diseases, Drug screening, Infection

## Abstract

To discover new drugs to combat COVID-19, an understanding of the molecular basis of SARS-CoV-2 infection is urgently needed. Here, for the first time, we report the crucial role of cathepsin L (CTSL) in patients with COVID-19. The circulating level of CTSL was elevated after SARS-CoV-2 infection and was positively correlated with disease course and severity. Correspondingly, SARS-CoV-2 pseudovirus infection increased CTSL expression in human cells in vitro and human *ACE2* transgenic mice in vivo, while *CTSL* overexpression, in turn, enhanced pseudovirus infection in human cells. CTSL functionally cleaved the SARS-CoV-2 spike protein and enhanced virus entry, as evidenced by CTSL overexpression and knockdown in vitro and application of CTSL inhibitor drugs in vivo. Furthermore, amantadine, a licensed anti-influenza drug, significantly inhibited CTSL activity after SARS-CoV-2 pseudovirus infection and prevented infection both in vitro and in vivo. Therefore, CTSL is a promising target for new anti-COVID-19 drug development.

## Introduction

The recent outbreak of novel coronavirus (SARS-CoV-2) disease (COVID-19) has imposed a severe public health burden worldwide. To prevent and treat this disease, effective methods, such as vaccines and drugs, are urgently needed. Although vaccination is underway in some countries, the impact of SARS-CoV-2 mutations and the degree of protection that a vaccine could have are some of the main issues under debate.^1^ On the other hand, the drug is more convenient and acceptable to people. Unfortunately, there is currently no efficient drug that can be used. Thus, the United States Food and Drug Administration (FDA) has approved remdesivir for the treatment of COVID-19 under an emergency use authorization. It is believed that remdesivir may be useful but not universally effective.^2^ Therefore, broadening the spectrum of therapeutic targets is important.

Both SARS-CoV-1, which emerged in 2002, and the novel SARS-CoV-2 infect host cells through binding of their viral envelope spike (S) proteins to the same receptor, angiotensin-converting enzyme 2 (ACE2).^3^ Our group focused on the correlation between ACE2 and metabolic diseases since the outbreak of SARS-CoV-1 infection in 2003. Our previous study revealed that ACE2 gene is highly associated with diabetes^4^; loss of ACE2 leads to a decrease in insulin secretion, as well as a progressive impairment of glucose tolerance^5,6^; ACE2 pathway can ameliorate local blood flow, inflammation, stress state, and fibrosis, which leads to the improvement of glucose transport, lipolysis, and adipokines production.^7–9^ These findings may partly explain the higher morbidity and mortality in COVID-19 patients combined with diabetes.^10^

To gain entry into host cells, diverse viruses depend on cleavage and activation of the S protein by host cell proteases.^11–14^ However, recent studies have focused primarily on furin and transmembrane protease serine 2 (TMPRSS2).^3,14^

Cathepsin L (CTSL), a member of the lysosomal cysteine protease, contains an L domain of α-helix and an R domain of β-sheet in the spatial structure.^15^ Its main function is proteolysis of protein antigens produced by pathogen endocytosis.^16^ Previous studies have indicated that membrane fusion of SARS-CoV-1 relies on proteolysis of the S protein by host CTSL in vitro.^17,18^ Therefore, it is reasonable to hypothesize that CTSL may be a target for the development of drugs to treat SARS-CoV-2 infection.^19,20^ This study, for the first time, analyzed circulating levels of CTSL in patients with COVID-19 and systematically studied the therapeutic effects of CTSL on this disease. It indicated that CTSL could be a promising therapeutic target for the prevention and treatment of COVID-19.

## Results

### Enrollment and characteristics of patients with COVID-19

A total of 108 consecutive COVID-19 inpatients admitted to Beijing Youan Hospital, Capital Medical University between January 21 and April 30, 2020, were investigated. After excluding 5 patients who were less than 18 years old, 1 pregnant patient, 8 patients who died in a very short time, and 7 patients who were unwilling to participate, 87 patients were ultimately included in the final analysis. Of these 87 patients, 67 had the nonsevere disease (2 mild, 65 moderate) and 20 had severe disease (15 severe, 5 critical) based on the severity classification established by the National Health Committee of China. Detailed demographic and clinical characteristics of the patients with COVID-19 are shown in Supplementary Table 1. The marked increases in aspartate aminotransferase (AST), creatine kinase MB (CK-MB), and myoglobin levels indicated that SARS-CoV-2 attacks liver and cardiac cells, consistent with previous reports.^21,22^

We also enrolled 125 healthy volunteers who were age- and sex-matched with the COVID-19 patients. Detailed demographic and clinical characteristics of the healthy individuals are shown in Supplementary Table 2. The comparative analysis of the parameters between COVID-19 patients and healthy individuals is shown in Supplementary Table 3.

### The circulating level of CTSL is elevated in patients with COVID-19

**Fig. 1:**
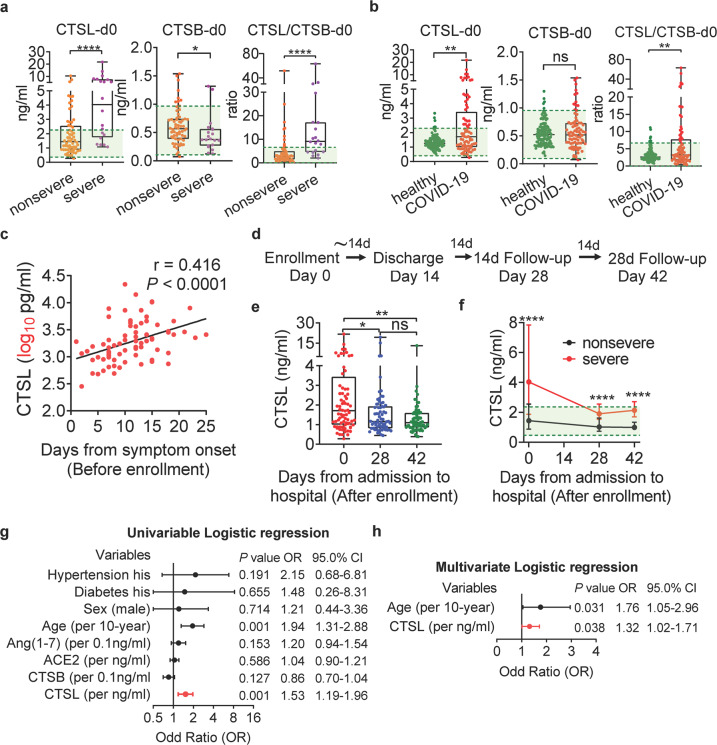
Circulating CTSL is elevated in patients with COVID-19. A total of 87 patients with COVID-19, including 20 with severe disease and 67 with nonsevere disease, and 125 healthy volunteers were enrolled in this study. **a** Plasma CTSL, CTSB, and CTSL/CTSB levels patients with severe (*n* = 20) and nonsevere COVID-19 (*n* = 67) upon hospital admission (day 0). Statistical significance was assessed by the Mann–Whitney *U* test (two-sided). **b** Plasma CTSL, CTSB and CTSL/CTSB levels in COVID-19 patients (day 0) (*n* = 87) and age-/sex-matched healthy volunteers (*n* = 125). The green lines in panels **a** and **b** indicate the reference ranges for each parameter, established as the mean values ± 2 SD in the healthy participants. Statistical significance was assessed by the Mann–Whitney *U* test (two-sided). **c** Correlation between CTSL in plasma from COVID-19 patients (*n* = 87) and the number of days from symptom onset to blood collection before therapy. Statistical significance was assessed by Spearman correlation analysis (two-sided). **d** Flowchart of the follow-up study. Patients were admitted to the hospital on day 0 and experienced a mean hospitalization time of 14 days (day 14). Then, they were followed up on the 14th day (day 28) and the 28th day (day 42) after discharge from the hospital. Blood samples were collected on days 0, 28, and 42. **e** CTSL levels in plasma from COVID-19 patients (*n* = 87) on days 0, 28, and 42 after enrollment. Statistical significance was assessed by the Kruskal–Wallis test with Dunn’s post hoc test. **f** Comparison of plasma CTSL levels between patients with nonsevere (*n* = 67) and severe COVID-19 (*n* = 20) on days 0, 28, and 42. The data are shown as the medians ± interquartile ranges. Statistical significance was assessed by the Mann–Whitney *U* test (two-sided). **g** Summary forest plot of candidate predictor variables associated with the severity of COVID-19 (*n* = 87) by univariable logistic regression. **h** Summary forest plot of candidate predictor variables associated with COVID-19 severity (*n* = 87) in multivariable logistic regression. The predictor variables used in the final model were hypertension, diabetes, sex, age, Ang(1–7), ACE2, CTSB and CTSL

As SARS-CoV-2 shares 79.6% sequence identity with SARS-CoV-1,^23^ we measured the plasma levels of several proteins that are essential to SARS-CoV-1 entry into host cells. ACE2 is the entry receptor for both SARS-CoV-1 and SARS-CoV-2. The endosomal cysteine proteases CTSL and cathepsin B (CTSB) mediate cleavage of the SARS-CoV-1 S protein, which is necessary for entry of coronavirus into host cells.^17,24^ Therefore, plasma levels of CTSL, CTSB, ACE2, and its products (angiotensin 1–7 (Ang(1–7)), were measured in patients with COVID-19. Plasma levels of Ang(1–7) were slightly lower in patients with severe disease than in patients with nonsevere disease, while plasma levels of ACE2 were the same between the two groups (Supplementary Table 4). Interestingly, the plasma level of CTSL and CTSL/CTSB were markedly higher, while that of CTSB was slightly lower, in patients with severe disease than in patients with nonsevere disease (Fig. 1a and Supplementary Table 4).

To further confirm the correlation between changes in cathepsin levels and COVID-19, plasma CTSL and CTSB levels were measured in the 125 healthy volunteers, and the reference ranges for each parameter were established as the mean values ± 2 SD in the healthy participants, indicated by the green boxes in the figures (Fig. 1a, b). The CTSL and CTSL/CTSB levels were markedly higher in patients with COVID-19 than in healthy volunteers, while the CTSB level was unchanged in the patients (Fig. 1b). CTSL and CTSL/CTSB increased in COVID-19 patients than in healthy individuals, and in severe patients than in nonsevere patients. These results indicated that CTSL or CTSL/CTSB were associated with the SARS-CoV-2 infection. Further investigations and validations are needed.

### The circulating level of CTSL changes with the course of COVID-19

Notably, a strong correlation was found between the CTSL level and the number of days from symptom onset to blood collection before therapy (Fig. 1c). We further performed a follow-up study to determine the correlation between the CTSL level and COVID-19. Patients with COVID-19 experienced a mean hospitalization time of 14 days (day 14) and were followed up on the 14th day (day 28) and 28th day (day 42) after hospital discharge (Fig. 1d). In the follow-up study, the elevated levels of CTSL were dramatically decreased on day 28 and remained at a stable level on day 42 (Fig. 1e). However, the difference between the severe and nonsevere groups persisted for 42 days after admission to the hospital, although in most patients, the CTSL level returned to the normal range after discharge (Fig. 1f). These results indicated that CTSL was significantly associated with SARS-CoV-2 infection.

### CTSL is an independent factor for severity in patients with COVID-19

The correlations between disease severity and clinical parameters, including CTSL, CTSB, ACE2, Ang(1–7), age, sex, and coexistence of diabetes or hypertension, were estimated using Spearman’s rho correlation coefficient. Severity was positively correlated with CTSL and age but was negatively correlated with CTSB. In addition to being correlated with severity, CTSL was also positively correlated with age and history of hypertension but was negatively correlated with CTSB (Supplementary Table 5). In univariable logistic regression analysis, the odds ratio (OR) of experiencing critical condition was significantly higher in patients with a higher CTSL level (OR, 1.53 per ng/ml; 95% CI, 1.19–1.96; *P* = 0.001) and older age (OR, 1.94 per 10 years; 95% CI, 1.31–2.88; *P* = 0.001), while CTSB, ACE2, Ang(1–7), sex, and coexistence of diabetes or hypertension did not significantly contribute to the odds of experiencing critical condition (Fig. 1g). Multivariable logistic regression indicated that CTSL was an independent factor for severe disease status after adjustment for hypertension, diabetes, sex, age, Ang(1–7), ACE2, and CTSB (Fig. 1h). Taken together, these findings led us to conclude that CTSL was highly correlated with SARS-CoV-2 infection and associated with the severity of the disease.

### The CTSL level is elevated in SARS-CoV-2 pseudovirus-infected cells in vitro

As the CTSL level is significantly elevated in the plasma of patients with COVID-19, we speculated that CTSL may be an important biomarker and therapeutic target for COVID-19. To test this hypothesis, we conducted a series of in vitro and in vivo experiments using a SARS-CoV-2 pseudovirus system. This pseudovirus is composed of replication-defective vesicular stomatitis virus (VSV) particles (G*ΔG-VSV-based pseudovirus) bearing SARS-CoV-2 S proteins (SARS-2-S), faithfully reflects key aspects of SARS-CoV-2 cell entry^3^ as we previously reported^25^ and can be used safely in biosafety level 2 (BSL-2) laboratories (Supplementary Fig. 1a, b). VSV encodes several kinds of proteins, including host cell attachment glycoprotein (VSV-G) and phosphoprotein (VSV-P). For the G*ΔG-VSV-based pseudovirus system, the VSV-G gene was deleted and replaced with genes encoding firefly luciferase.^25^ The infected cells would therefore express VSV-P and luciferase proteins after they were infected by the G*ΔG-VSV-based pseudovirus. Accordingly, both the luciferase activity and the mRNA level of VSV-P can be used as indicators for pseudovirus infection (Supplementary Fig. 1c).

First, to determine the cell line with the highest susceptibility to SARS-2-S-driven entry, we compared several human cell lines, including human hepatoma cells (Huh7), human embryonic kidney cells (HEK293T), human lung adenocarcinoma cells (A549), and human lung adenocarcinoma cells (Calu-3) (Supplementary Fig. 2a). The luciferase assay results revealed that the Huh7 cell line had the highest susceptibility to SARS-2-S pseudovirus infection (Supplementary Fig. 2b–f). In addition, the marked increases in the circulating levels of alanine aminotransferase (ALT) and AST in COVID-19 patients compared with healthy volunteers observed in our study (Supplementary Tables 1–2 and Supplementary Fig. 3a, b) and the hepatocellular injury observed in patients with COVID-19 by others suggested that SARS-CoV-2 attacks hepatocytes as target cells.^22^ Therefore, Huh7 cells were selected for the subsequent experiments in this study.

**Fig. 2:**
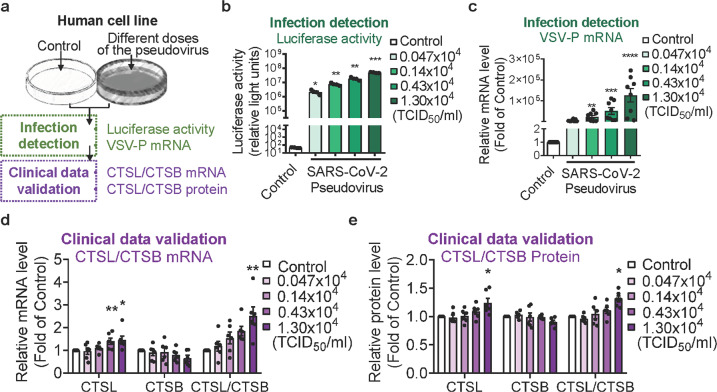
CTSL is elevated in SARS-CoV-2 pseudovirus-infected cells in vitro. **a** Schematic of the validation assay setup. Huh7 cells were infected with different doses of SARS-CoV-2 pseudovirus (from 0.047 × 10^4^ TCID_50_/ml to 1.30 × 10^4^ TCID_50_/ml). Cells not infected with pseudovirus were used as controls. Luciferase activity and VSV-P mRNA levels were measured to evaluate infection severity. The mRNA and protein levels of CTSL and CTSB in Huh7 cells were measured to validate the clinical data. **b**, **c** Luciferase activity (*n* = 4) (**b**) and VSV-P mRNA (*n* = 8) (**c**) levels increased dose-dependently 24 h after pseudovirus infection. Statistical significance was assessed by Brown–Forsythe and Welch’s ANOVA with Dunnett’s post hoc test in **b** by with the Kruskal–Wallis test with Dunn’s post hoc test for **c**. **d**, **e** Effects of SARS-CoV-2 pseudovirus infection on CTSL and CTSB mRNA levels (**d**) and protein levels (**e**). *n* = 6. Statistical significance was assessed by the Kruskal–Wallis test with Dunn’s post hoc test. Data are expressed as the mean ± s.e.m. values. *P < 0.05, **P < 0.01, ***P < 0.001, ****P < 0.0001

Next, to verify whether CTSL expression increases after SARS-CoV-2 infection in vitro, the protein and mRNA levels of CTSL and CTSB were measured in Huh7 cells infected with SARS-CoV-2 pseudovirus (Fig. 2a). Huh7 cells were infected with different doses of SARS-CoV-2 pseudovirus, as indicated by the luciferase activities (Fig. 2b) and VSV phosphoprotein (VSV-P) mRNA levels (Fig. 2c). Consistent with our clinical data, the mRNA (Fig. 2d) and protein (Fig. 2e) levels of CTSL increased in a dose-dependent manner after SARS-CoV-2 pseudovirus infection. These results confirmed our findings in patients with COVID-19 and indicated that SARS-CoV-2 infection caused CTSL upregulation.

### CTSL knockdown or overexpression affects pseudovirus infection in vitro

**Fig. 3:**
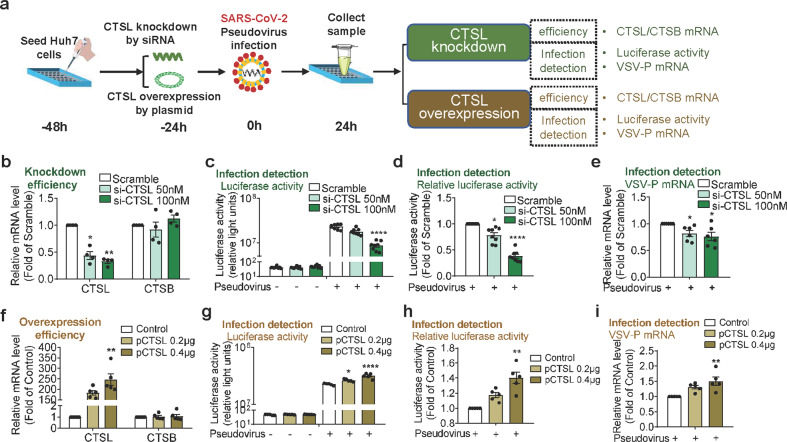
CTSL knockdown or overexpression affects pseudovirus infection in vitro. **a** Schematic of the CTSL knockdown and overexpression assay setup. **b** Dose-dependent knockdown of *CTSL* by siRNAs without affecting *CTSB* expression. *n* = 4. Statistical significance was assessed by the Kruskal–Wallis test with Dunn’s post hoc test. **c**–**e** Knockdown of *CTSL* dose-dependently inhibited SARS-2-S-driven entry, as measured by a luciferase assay and shown as absolute luciferase activity (*n* = 8) (**c**) and relative luciferase activity (*n* = 8) (**d**) values, and VSV-P mRNA levels (*n* = 6) (**e**). Statistical significance was assessed by the Kruskal–Wallis test with Dunn’s post hoc test. **f** Dose-dependent overexpression of *CTSL* with a plasmid encoding the *CTSL* gene without affecting *CTSB* expression (*n* = 5). Statistical significance was assessed by the Kruskal–Wallis test with Dunn’s post hoc test. **g**–**i** Overexpression of *CTSL* dose-dependently promoted SARS-2-S-driven entry, as measured by a luciferase assay and shown as absolute luciferase activity (**g**) and relative luciferase activity **h**, values, and VSV-P mRNA levels (**i**) *n* = 5. Statistical significance was assessed by the Kruskal–Wallis test with Dunn’s post hoc test. The data are expressed as the mean ± s.e.m. values. *P < 0.05, **P < 0.01, ***P < 0.001, ****P < 0.0001

To investigate whether CTSL is required for cell entry of SARS-CoV-2, we used siRNAs against human *CTSL* (si-CTSL) and plasmids encoding human *CTSL* (pCTSL) to knockdown and overexpress the *CTSL* gene in Huh7 cells, respectively. si-CTSL treatment dose-dependently downregulated *CTSL* without affecting *CTSB* expression at both the mRNA and the protein level (Fig. 3b and Supplementary Fig. 4a). Knockdown of *CTSL* led to a significant dose-dependent reduction in pseudovirus cell entry, as evidenced by the luciferase activity and VSV-P mRNA level (Fig. 3c–e). In contrast, overexpression of *CTSL* markedly increased pseudovirus cell entry in a dose-dependent manner without affecting *CTSB* expression at both the mRNA and the protein levels (Fig. 3f–i and Supplementary Fig. 4b). All these results suggested that CTSL was critical for SARS-CoV-2 infection.

### CTSL cleaves the S protein, and this cleavage promotes cell–cell fusion

**Fig. 4:**
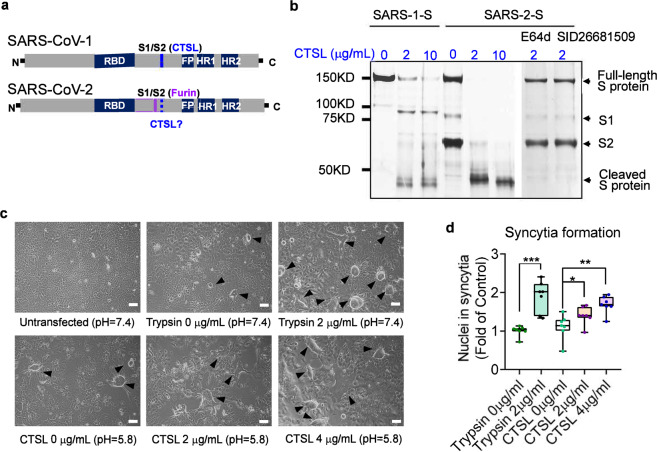
CTSL cleaves the SARS-CoV-2 spike (S) protein, and this cleavage promotes cell–cell fusion. **a** Overview of the SARS-CoV-1 and SARS-CoV-2 S1/S2 cleavage sites. FP (fusion peptide), HR1 (heptad repeat 1), and HR2 (heptad repeat 2) are units of the S2 subunit that function in membrane fusion. **b** Analysis of CTSL-mediated S-protein cleavage. Purified SARS-CoV-1 or SARS-CoV-2 S protein was incubated in the presence or absence (assay buffer, pH = 5.5) of CTSL (2 or 10 μg/ml in assay buffer, pH = 5 .5) at 37 °C for 1 h. The reaction system of 2 μg/ml CTSL was further supplemented with CTSL inhibitors (20 μM E64d or 20 μM SID 26681509), as indicated. Proteins were subjected to SDS-PAGE and detected by silver staining. Representative data from three independent experiments are shown. **c** Syncytium-formation assay: Huh7 cells were untransfected (Null) or transfected with plasmid to express the SARS-CoV-2 S protein. Cells were incubated in the presence or absence (PBS, pH = 7.4) of trypsin (2 μg/ml in PBS, pH = 7.4) or in the presence or absence (PBS, pH = 5.8) of CTSL (2 or 4 μg/ml in PBS, pH = 5.8) for 20 min. Images were acquired after an additional 16 h incubation in the medium. (scale bars, 50 μm). The black arrowheads indicate syncytia. Representative data from seven independent experiments are shown. **d** Quantitative analysis of syncytia in panel **c**. *n* = 7. Statistical significance was assessed by one-way ANOVA with Tukey’s post hoc test. The data are expressed as the mean ± s.e.m. values. *P < 0.05, **P < 0.01, ***P < 0.001

CTSL cleaves the SARS-CoV-1 S protein into S1 and S2 subunits and proteolytically activates cell–cell fusion.^17^ Unlike the SARS-CoV-1 S protein, the SARS-CoV-2 S protein is precleaved by the proprotein convertase furin at the S1/S2 cleavage site (Fig. 4a). Hence, the effects of CTSL in SARS-CoV-2 seem to be replaced by those of furin. However, no report has examined the effects of CTSL on SARS-CoV-2 S-protein cleavage and the function of CTSL in cell–cell fusion. Here, we directly detected the cleavage of purified SARS-CoV-2 S protein by CTSL. Treatment with CTSL resulted in successful cleavage of purified SARS-CoV-1 S protein, suggesting that the experimental system was feasible. Notably, CTSL also efficiently cleaved purified SARS-CoV-2 S protein in a dose-dependent manner (Fig. 4b). To further confirm the specificity, CTSL inhibitors were employed. Given that no currently available drug specifically inhibits CTSL,^26^ two compounds that have been demonstrated to have inhibitory activity against CTSL (E64d, a broad-spectrum cathepsin inhibitor, and SID26681509, a relatively selective CTSL inhibitor) were used. The cleavage activity of CTSL was blocked by E64d and SID 26681509 (Fig. 4b). These results indicated that CTSL efficiently cleaved the SARS-CoV-2 S protein into smaller fragments after its initial cleavage by furin.

To investigate whether CTSL functionally cleaves the SARS-CoV-2 S protein, we performed a cell–cell fusion assay by recording SARS-2 S-protein-driven formation of multinucleated giant cells (syncytium). No syncytia were observed without S-protein expression, while SARS-2-S expression alone (treated with PBS, pH = 7.4) resulted in syncytium formation. Trypsin treatment (2 μg/ml in PBS, pH = 7.4), which has been shown to induce S-protein-mediated cell–cell fusion,^24,27^ markedly increased syncytium formation nearly twofold. In addition, compared to mock treatment (PBS, pH = 5.8), CTSL (in PBS, pH = 5.8) dose-dependently induced an increase in syncytium formation of up to ~70%. These data indicated that CTSL activity, not acidic conditions, was responsible for the increase in syncytium formation (Fig. 4c, d). Therefore, these results led us to conclude that CTSL efficiently cleaved the SARS-CoV-2 S protein and that this cleavage promoted S-protein-mediated cell–cell fusion.

### CTSL inhibitors prevent SARS-CoV-2 pseudovirus infection in vitro

**Fig. 5:**
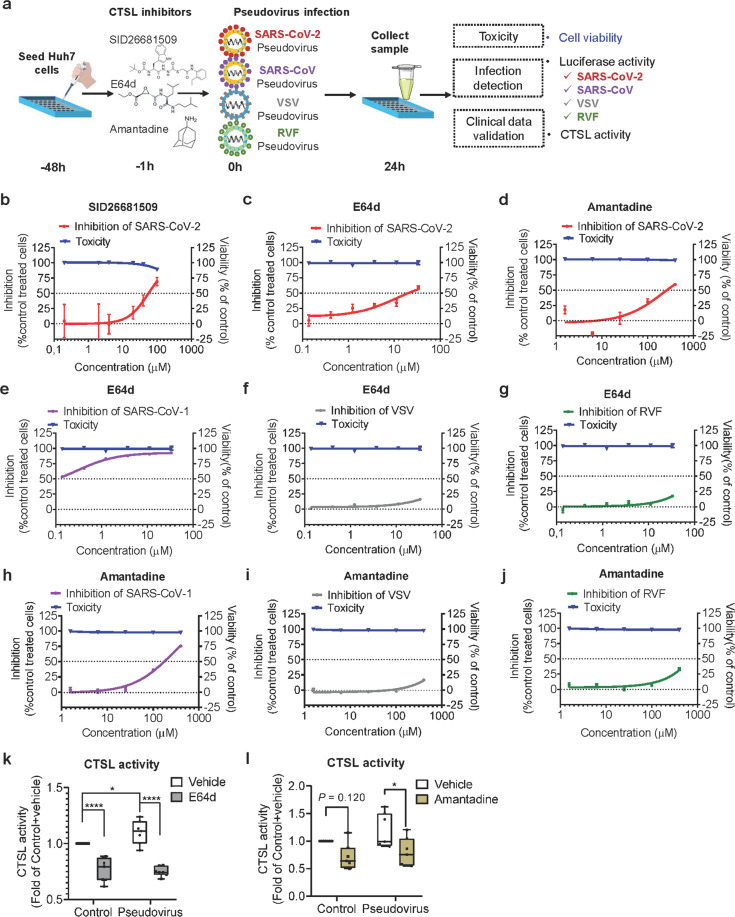
CTSL inhibitors block SARS-2-S-driven cell entry in vitro. **a** Schematic of the CTSL inhibitor assay setup. Huh7 cells were pretreated with different drugs 1 h before infection with different pseudoviruses, as indicated, at the same dose (1.3 × 10^4^ TCID_50_/ml). Pseudovirus infection and cell viability were evaluated by luciferase activity and MTT assay, respectively. **b**–**d** Inhibition of SARS-2-S-driven cell entry by different doses of SID 26681509 (**b**), E64d (**c**), and amantadine (**d**) and viability of cells treated with different doses of the drugs as indicated. *n* = 4. **e**–**g** Effects of E64d on SARS-CoV-1 pseudovirus (**e**), VSV pseudovirus (**f**), and RVF pseudovirus (**g**) infection and viability of cells treated with different doses of E64d as indicated. *n* = 4. **h**–**j** Effects of amantadine on SARS-CoV-1 pseudovirus (**h**), VSV pseudovirus (**i**), and RVF pseudovirus (**j**) infection and viability of cells treated with different doses of amantadine as indicated. *n* = 4. **k**, **l** Effects of drug pretreatment on CTSL enzyme activity in Huh7 cells with or without pseudovirus infection. Huh7 cells were pretreated with vehicle, 30 μM E64d (**k**) or 300 μM amantadine (**l**) for 1 h and were then infected with SARS-CoV-2 pseudovirus at a dose of 1.3 × 10^4^ TCID_50_/ml. Cells not infected with pseudovirus were used as controls. *n* = 7. Statistical significance was assessed by two-way ANOVA with the Holm–Sidak post hoc test for multiple comparisons. The data are expressed as the mean ± s.e.m. values. *P < 0.05, **P < 0.01, ***P < 0.001, ****P < 0.0001

To further confirm the role of CTSL in SARS-CoV-2 infection, Huh7 cells were treated with CTSL inhibitors, as shown in Fig. 5a. Both SID 26681509 and E64d significantly inhibited SARS-CoV-2 pseudovirus infection. As E64d exhibited less cytotoxicity than SID 26681509, it was selected for the subsequent experiments (Fig. 5b, c).

Furthermore, we were interested to find that amantadine, a prophylactic agent approved by the US FDA in 1968 for influenza and later for Parkinson’s disease, has been reported to suppress the gene transcription of *CTSL*.^19^ We next examined the impact of amantadine on SARS-CoV-2 infection and found that it significantly inhibited pseudovirus infection with little cytotoxicity (Fig. 5d). Moreover, both E64d and amantadine also significantly prevented SARS-CoV-1 S-protein-driven but not VSV-G protein-driven or Rift Valley fever (RVF) virus (a member of the bunyavirus family that does not require CTSL for cell entry)^28,29^ G protein-driven pseudovirus infection (Fig. 5e–j).

Finally, CTSL enzyme activity was measured in Huh7 cells. Treatment with E64d (Fig. 5k) and amantadine (Fig. 5l) inhibited the enzyme activity and blocked the activation of CTSL induced by SARS-CoV-2 pseudovirus infection. These results indicated that the therapeutic effects of both E64d and amantadine were at least partially mediated by inhibition of CTSL enzyme activity.

### CTSL inhibitors prevent pseudovirus infection in humanized mice

**Fig. 6:**
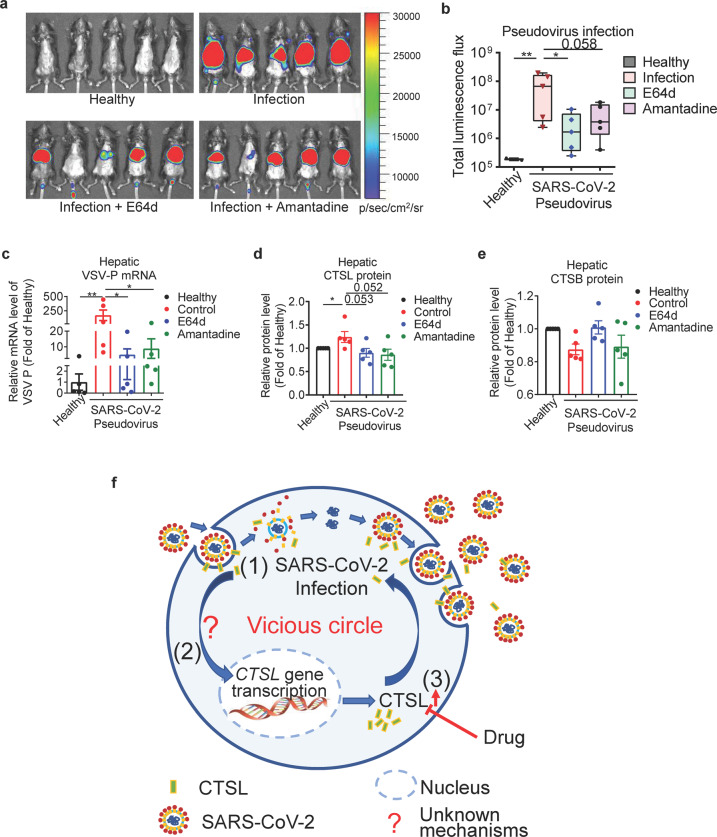
CTSL inhibitors prevent pseudovirus infection in humanized mice. Human *ACE2* transgenic mice were randomly divided into four groups and pretreated with vehicle or different drugs (E64d or amantadine) as indicated 2 days prior to virus inoculation via tail vein injection (1.5 × 10^6^ TCID_50_ per mouse). Mice without pseudovirus inoculation were used as the healthy control group. Bioluminescence was measured 1 day post infection and visualized in pseudocolor. **a** The relative intensities of emitted light are presented as the photon flux values in photon/(sec/cm^2^/sr) and displayed as pseudocolor images, with colors ranging from blue (lowest intensity) to red (highest intensity). **b** Pseudovirus infection in each group as indicated by the total flux values. Statistical significance was assessed by one-way ANOVA with Tukey’s post hoc test for multiple comparisons. **c** Pseudovirus infection as indicated by the liver VSV-P mRNA levels in each group. Statistical significance was assessed by one-way ANOVA with Tukey’s post hoc test for multiple comparisons. **d** Hepatic CTSL protein levels in each group. Statistical significance was assessed by the Kruskal–Wallis test with Dunn’s post hoc test. **e** Hepatic CTSB protein levels in each group. Statistical significance was assessed by the Kruskal–Wallis test with Dunn’s post hoc test. **f** Proposed mechanism of CTSL action in SARS-CoV-2 infection. (1) CTSL cleaves the SARS-2-S protein and releases the virus from the endosome. (2) SARS-CoV-2 promotes CTSL gene transcription and enzyme activity through unknown mechanisms. (3) Upregulation of CTSL, in turn, enhances SARS-CoV-2 infection. *n* = 5. The data are expressed as the mean ± s.e.m. values. *P < 0.05, **P < 0.01

To further verify whether CTSL inhibition can prevent pseudovirus infection in vivo, the effects of E64d and amantadine on preventing SARS-CoV-2 pseudovirus infection were assessed in mice by bioluminescence imaging (BLI). Because SARS-CoV-2 recognizes the human ACE2 protein but not the mouse or rat ACE2 as the cell entry receptor,^30^ *hACE2* humanized mice—model mice engineered to express *hACE2* via CRISPR/Cas9 knock-in technology, as we previously reported^31^—were employed. The *hACE2* humanized mice were randomly divided into four groups and treated with either vehicle or different drugs as indicated. Bioluminescence was measured and visualized in pseudocolor as an indicator of SARS-CoV-2 pseudovirus infection severity. Pseudovirus-infected humanized mice showed a significantly higher luminescence signal than healthy control mice, indicating that the mice were successfully infected (Fig. 6a, b). Compared to the vehicle treatment, E64d significantly prevented SARS-CoV-2 pseudovirus infection. Amantadine also showed suppressive effects on pseudovirus infection, but the differences were not statistically significant (*P* = 0.058) (Fig. 6b). The hepatic VSV-P mRNA level was markedly increased in humanized mice after SARS-CoV-2 pseudovirus infection, but this increase was significantly suppressed by pretreatment with either E64d or amantadine (Fig. 6c), indicating that both drugs indeed prevented SARS-CoV-2 pseudovirus infection.

Notably, the protein level of CTSL in the liver was significantly increased in SARS-CoV-2-infected mice; this increase was reversed by treatment with either E64d or amantadine (Fig. 6d), while the CTSB level was not significantly affected (Fig. 6e). The lungs were slightly infected by SARS-CoV-2 pseudovirus. Accordingly, the trend of CTSL elevation was present, but the differences were not statistically significant (Supplementary Fig. 5a, b).

## Discussion

Diverse viruses, including SARS-CoV-1, have been shown to affect the expression of host cell infection-related genes.^32–34^ Here, for the first time, we found that SARS-CoV-2 infection promoted the gene expression of *CTSL* both in vivo and in vitro, while *CTSL* overexpression, in turn, enhanced pseudovirus infection in human cells. The detailed mechanisms of this “vicious circle” remain to be investigated. Interestingly, a recent study also found that SARS-CoV-2 can exploit interferon-driven upregulation of ACE2 to enhance infection,^35^ suggesting that the mechanistic involvement of other infection-related genes in SARS-CoV-2 infection requires further exploration. However, CTSL would be a promising therapeutic target for inhibitors that could not only inhibit entry of the virus but also block the vicious circle (Fig. 6f).

Infection of cells with many kinds of viruses depends on specific host cell proteases.^36,37^ A recent study suggested that CTSL might be involved in SARS-CoV-2 entry into HEK293 cells in vitro.^27^ However, clinical evidence of the role of CTSL in SARS-CoV-2 infection is lacking. First, no investigation of circulating levels of CTSL in patients with COVID-19 had been reported before this study. Second, tissue expression of CTSL has not yet been investigated in SARS-CoV-2 infection, although circulating levels of CTSL can reflect its expression profile in many organ tissues, such as vascular tissues.^38^ In this study, we first analyzed the circulating level of CTSL in patients with COVID-19 and found that it precisely reflected the severity and status of COVID-19. This relationship may be attributed to the elevated expression of CTSL observed in pseudovirus-infected human cells and mouse livers.

Previous studies have suggested that CTSL inhibitors effectively prevent the infection of many other coronaviruses, including SARS-CoV-1, Middle East respiratory syndrome coronavirus (MERS-CoV), and human coronavirus (HCoV)-229E.^39–41^ CTSL is thought to be a potential target for the treatment of COVID-19,^19,20^ although no systematic study has been conducted. Previous studies have indicated that furin and TMPRSS2 also play crucial roles in SARS-CoV-2 infection.^3,14^ In this study, we showed that CTSL functionally cleaved the SARS-CoV-2 S protein into smaller fragments. This cleavage resulted in an up to ~70% enhancement in S-protein-mediated cell–cell fusion. CTSL efficiently enhanced SARS-CoV-2 infection, as evidenced by our overexpression and knockdown data in vitro and inhibition data in vivo. Besides, furin and TMPRSS2 cleave the SARS-CoV-2 S protein at different cleavage sites.^14,42^ Our results indicated that the CTSL cleavage site was different from that of furin. TMPRSS2 also has been reported to mediate ACE2 activation in SARS-CoV-1 infection.^43^ Therefore, it is reasonable to conclude that CTSL, TMPRSS2, and furin are all required for SARS-CoV-2 infection. Broadening the range of therapeutic targets for COVID-19 is important, and the effects of CTSL should not be underestimated.

The COVID-19 pandemic has motivated the most immense efforts to date to identify drugs that can safely, quickly, and effectively reduce morbidity and mortality. Focusing on repurposing a licensed drug for COVID-19 may be more efficient than starting with a preclinical drug. Thus, repurposing of many FDA-approved drugs for COVID-19 has been suggested, e.g., as was accomplished with the antimalarial drugs chloroquine (CQ) and hydroxychloroquine (HCQ) for rheumatoid arthritis and with the anti-influenza drug amantadine for Parkinson’s disease.^44–46^

Amantadine is a preventive agent first used for influenza and later for Parkinson’s disease. Only three observational clinical case reports with small numbers of patients (*n* ≤ 15 for all) speculated that amantadine may be used for COVID-19 treatment, no systematic clinical trial in a human population was performed.^47–49^ Some other papers only raised a hypothesis but without any validation experiment.^50,51^ In this study, we systematically investigated the role of amantadine in the treatment of COVID-19 and found that amantadine inhibited CTSL enzyme activity in the setting of SARS-CoV-2 pseudovirus infection. Amantadine significantly inhibited SARS-CoV-1 and SARS-CoV-2 cell entry with little cytotoxicity. Moreover, amantadine showed anti-pseudovirus effects, with downregulation of CTSL expression, in humanized mice. Therefore, amantadine may be a potent therapeutic drug.

In conclusion, we report that SARS-CoV-2 infection promoted CTSL expression and enzyme activity, which, in turn, enhanced viral infection. CTSL functionally cleaved the SARS-CoV-2 S protein and enhanced viral entry. Hence, CTSL is likely an important therapeutic target for COVID-19. Furthermore, we showed that amantadine, a licensed anti-influenza drug, significantly inhibited CTSL activity after SARS-CoV-2 pseudovirus infection and prevented infection both in vitro and in vivo. Therefore, our study shows that amantadine or other CTSL inhibitors may be a potential therapeutic strategy to SARS-CoV-2 infection. In the future, experiments using live SARS-COV-2 viruses and clinical trials are needed to investigate the role of CTSL inhibitors in treating COVID-19.

## Materials and methods

### Ethical approval statement

The study was conducted with the approval of the Ethics Committee of Beijing Youan Hospital, Capital Medical University and the Ethics Committee of Beijing Tongren Hospital, Capital Medical University.

### Participants and clinical samples

Patients diagnosed with COVID-19 and hospitalized in Beijing Youan Hospital, Capital Medical University from January 21 to April 30, 2020, were enrolled in this study. All enrolled patients were confirmed positive for SARS-CoV-2 nucleic acid by real-time polymerase chain reaction (RT-PCR). The RT-PCR assay was conducted as per the protocol established by the World Health Organization (WHO). The diagnosis and clinical classification criteria and treatment plan (version 7.0) of COVID-19 were launched by the National Health Committee of China (http://www.nhc.gov.cn/). The clinical classification of severity is as follows: (1) Mild, having only mild symptoms, imaging shows no pneumonia. (2) Moderate, with fever, respiratory tract symptoms, and imaging shows pneumonia. (3) Severe, meet any of the following signs: respiratory distress, respiratory rate ≥30 beats/min; b in the resting state, finger oxygen saturation ≤93%) arterial blood oxygen partial pressure (PaO_2_/oxygen concentration (FiO_2_) ≤300 mmHg (1 mmHg = 0.133 kPa). (4) Critical, one of the following conditions: a respiratory failure occurs and requires mechanical ventilation; b Shock occurs; c ICU admission is required for combined organ failure. Patients with COVID-19 experienced a mean of 14 days of hospitalization (day 14) and were followed up on the 14th day (day 28) and 28th day (day 42) after discharge from the hospital. Blood samples were collected shortly after the admission to the hospital (day 0) (for some patients who were transferred from other hospitals, their blood samples were collected shortly after admission at Beijing Youan hospital) and on days 28 and 42.

Demographic, clinical, and laboratory data were extracted from the electronic hospital information system using a standardized form.

A total of 125 sex- and age-matched healthy volunteers were recruited in Beijing Tongren Hospital, Capital Medical University. The including criteria are as following: (1) Age 18–70 y; (2) no underlying diseases; (3) no long-term use of any medication; (4) willing to participate in the study. Blood samples were collected after overnight fasting for the determination of biochemical parameters, CTSL and CTSB concentrations. All biochemical measurements have participated in the Chinese Ministry of Health Quality Assessment Program.

This study was conducted with the approval of the Ethics Committee of Beijing Youan Hospital, Capital Medical University and the Ethics Commission waived the requirement for informed consent.

### Membrane fusion biomarkers of the virus

Plasma samples of patients with COVID-19 collected at admission (d0), 14th day after discharge (d28), and 28th day after discharge (d42) were collected and stored at −80 °C within 2 h. The samples were analyzed using commercially available enzyme-linked immunosorbent assays (ELISA) following the manufacturer’s instructions. All samples were detected without virus inactivation to retain the original results in a P2 + biosafety laboratory. Angiotensin (1–7) and ACE2 were measured using the Human Angiotensin (1–7) and Human ACE2 Elisa kit (Cloud-Clone Corp, Houston, Texas, USA) with detection limits of <10 and 0.64 pg/ml, respectively. CTSL and CTSB were measured using the Human CTSL and Human CTSB ELISA kit (Elabscience, Houston, TX, USA) with detection limits of 37.5 pg/ml and 0.1 ng/ml, respectively. The kits were designed for usage with human serum or plasma samples and showed no cross-reactions.

### Experimental mice

The study used 4–5-week-old human ACE2 transgenic mice, a mouse model expressing human ACE2 (hACE2) generated by using CRISPR/Cas9 knock-in technology as previously reported.^31^ The hACE2 mice used in this manuscript were 4–5-weeks-old male C57BL/6 mice, with the body weight between 13 and 17 g. And the mice were validated in our previous paper.^31^ All animal protocols were approved by the Ethical Review Committee at the Institute of Zoology, Capital Medical University, China.

### Cell culture and reagents

The human hepatoma cell line Huh7, human lung adenocarcinoma A549 cells, and human HEK293T(293T) cells were maintained in high glucose Dulbecco’s modified Eagle’s medium (DMEM) (Sigma-Aldrich, St. Louis, MO, USA) supplemented with 10% fetal bovine serum (FBS, Gibco, Carlsbad, CA), 100 units/ml penicillin and 100 mg/ml streptomycin (Thermo Fisher Scientific). The human Calu-3 lung adenocarcinoma cell line was cultured in minimum essential medium (Eagle) with 2 mM l-glutamine and Earle’s BSS adjusted to contain 1.5 g/l sodium bicarbonate, 0.1 mM non-essential amino acids and 1.0 mM sodium pyruvate and 10% FBS. All the cells were maintained at 37 °C in a humidified atmosphere containing 95% air and 5% CO_2_. E64d (Cat. No. HY-100229), SID 26681509 (Cat. No. HY-103353), and Amantadine (Cat. No. HY-B0402A) were purchased from Med Chem Express (MCE, NJ, USA). Human CTSL expression plasmid (pENTER-CTSL, Cat. No. CH807099, Pubmed ID: NM_001912), and pENTER-vector (Cat. No. PD88001) were purchased from Vigenebio Ltd (China).

### Pseudovirus

The SARS-CoV-2, SARS-CoV-1, rift valley fever (RVF), and vesicular stomatitis virus (VSV) pseudovirus were generated with the incorporation of SARS-CoV-2 spike protein (SARS-2-S), SARS-CoV-1 spike protein (SARS-1-S), RVF host cell attachment glycoprotein (RVF-G), and VSV host cell attachment glycoprotein (VSV-G) into VSV-based pseudovirus system. The pseudoviruses used in the current study have been validated in previous studies.^52–54^ For this VSV-based pseudovirus system, the backbone was provided by VSV-G pseudotyped virus (G*ΔG-VSV) that packages expression cassettes for firefly luciferase instead of VSV-G in the VSV genome.^25^ Therefore, the luciferase activity and the mRNA level of VSV phosphoprotein (VSV-P) were used for indicators of pseudovirus infection.

### Luciferase assay

The activities of firefly luciferases were measured on cell lysates using luciferase substrate (Perkinelmer, BRITELITE PLUS 100 ml KIT, Cat. No. 6066761) following the manufacturer’s instructions. Briefly, for 96-well plates, the culture supernatant was aspirated gently to leave 100 μl in each well; then, 100 μl of luciferase substrate was added to each well. Two minutes after incubation at 37 °C, 150 μl of lysate was aspirated to a clean 1.5 ml sterile EP tube to measuring the firefly luciferase activity for each well rapidly using a luminometer (Turner BioSystems) as described previously.^55^

### Cell line selection

Huh7, 293T, A549, and Calu-3 cells were plated in 48-well plates, respectively, and infected with different doses of SARS-CoV-2 pseudovirus (starting from 0 to 1.3 × 10^4^TCID_50_/ml). The cells were cultured for another 24 h before luciferase activity analysis. Cells without the addition of pseudovirus as the cell control. The most susceptible cell line was selected for subsequent experiments.

### Clinical data verification in vitro

To verify the clinical data, Huh7 cells were plated in 48-well plates and allowed to adhere until the cells are about 70% confluent, followed by infecting with different doses of SARS-CoV-2 pseudovirus (starting from 0 to 1.3 × 10^4^TCID_50_/ml). After 24 h incubation, the cells were lysed for analysis of the firefly luciferase activity, VSV-P mRNA, and the detection of CTSL and CTSB by ELISA assays.

### Cleavage of SARS-CoV-2 S protein by CTSL

The purified extracellular domain of SARS-CoV-2 S protein (YP_009724390.1. Sino Biological, Cat. No. 40589-V08B1) and SARS-CoV S protein (NP_828851.1. Sino Biological, Cat. No. 40634-V08B) were purchased from Sino Biological (China). One microgram of each protein was incubated with 2 or 10 μg/ml CTSL (Sigma-Aldrich, Cat. No. SRP0291) in assay buffer (400 mM sodium acetate, pH 5.5, with 4 mM EDTA and 8 mM DTT) for 1 h at 37 °C. CTSL was preactivated in 30 °C for 1 min before use. Where indicated, 20 μM E64d or 20 μM SID 26681509 was added in the reaction system with 0.5 μg SARS-CoV-2 S protein and 2 μg/ml CTSL. The proteins were then subjected to sodium dodecyl sulfate-polyacrylamide gel electrophoresis (SDS-PAGE) and analyzed by silver stain (Thermo Fisher Scientific, Cat. No. 24612).

### Syncytium-formation assay

Huh7 cells were seeded in 24-well plates and transfected with SARS-CoV-2 S-protein expression plasmids (Sino Biological, Cat. No. VG40589-CF) (0.65 μg/well) using Lipofectamine 3000 reagent (Thermo Fisher Scientific). The transfection solutions were changed to standard culture medium 6 h post transfection and cells incubated for an additional 12 h. Next, cells were treated in the absence (PBS, pH = 7.4) or presence of 2 μg/ml trypsin (Sigma-Aldrich) (in PBS, pH = 7.4), or in the absence (PBS, pH = 5.8) or presence of 2 or 4 μg/ml CTSL (Sigma-Aldrich) (in PBS, pH = 5.8) for 20 min at 37 °C. Then, the solutions were changed to a standard culture medium, and the cells further incubated for 16 h. The pictures were captured under bright-field microscopy (Olympus) and analyzed the formation of syncytia by counting the nuclei in syncytia in five random microscopic fields.

### CTSL knockdown by siRNA and overexpression by plasmid in vitro

For CTSL knockdown, Huh7 cells were plated in 48-well plates, and transfected with 50 nM or 100 nM siRNAs against homo CTSL mRNA (si-CTSL) or 50 nM negative control siRNA (scramble) using Lipofectamine 3000 reagent. For CTSL overexpression, Huh7 cells were plated in 48-well plates and transfected with 0.2 μg or 0.4 μg human CTSL expression plasmid (pENTER-CTSL, pCTSL) or 0.2 μg control plasmid (pENTER-vector, Con). Twenty-four hours post transfection, the cells were lysed for analysis of the CTSL and CTSB mRNA levels to evaluate the efficiency of si-CTSL and pCTSL. To evaluate the effect of CTSL on SARS-CoV-2 entry, Huh7 cells were plated in 48-well plates, and transfected with si-CTSL or pCTSL under the same conditions stated above. Twenty-four hours post transfection, the medium was replaced with fresh medium. Then the cells were infected with SARS-CoV-2 pseudovirus (1.3 × 10^4^TCID_50_/ml) and cultured for another 24 h before firefly luciferase activity and VSV-P mRNA analysis. siRNA sequences were provided in Supplementary Table 6.

#### Effect of drug treatment on SARS-CoV-2 entry in vitro

The anti-SARS-CoV-2 activity of SID 26681509, E64d, and amantadine were performed in 96-well plates by quantification of the firefly luciferase activity. Huh7 cells were pretreated with different concentrations of drug or the equivalent amount of solvent for 1 h and then infected with SARS-CoV-2 pseudovirus (1.3 × 10^4^:TCID_50_/ml) in a 5% CO_2_ environment at 37 °C for 24 h before firefly luciferase activity analysis. In detail, the concentrations of different drugs as follow: SID 26681509 (0.2, 2, 4, 20, 40, and 100 μM), E64d (0.14, 0.42, 1.23, 3.7, 11.1, and 33.3 μM), and amantadine (1.56, 6.25, 25, 100, 400, and 1600 μM).

#### Cell viability assay

The effects of SID 26681509, E64d, and amantadine on cell viability were measured by MTT assay. Huh7 cells were seeded into a 96-well plate at a cell density of 0.5 × 10^4^ per well and allowed to adhere until the cells are about 70% confluent, followed by treatment with different concentrations of drugs or the equivalent amount of solvent for 24 h. The concentrations of different drugs were detailed above. Cells without any treatments as the blank control. After treatments, MTT was added into the culture medium to the final concentration of 0.5 mg/ml, and then the cells were incubated for 4 h at 37 °C in an incubator. After removing the culture medium, the cells were lysed by gently rotating in 200 μl DMSO for 10 min in darkness at room temperature. The absorbance at 570 nm was measured using an automatic plate reader. The average absorbance reflected cell viability with the data normalized to the blank control group. Experiments were done in quintuplicates and repeated at least three times.

#### Animal experiments

The anti-SARS-CoV-2 activity of E64d and amantadine were performed in human ACE2 transgenic mice by bioluminescent imaging (BLI) assay as before.^56^ Mice were treated with E64d (12.5 mg/kg body weight) or amantadine (50 mg/kg body weight) or the equivalent amount of solvent once a day via the intraperitoneal (IP) route 2 days prior to virus inoculation. Then mice were injected with 1.5 × 10^6^ TCID_50_ SARS-CoV-2 pseudovirus per mouse via tail vein injection (1 ml per mouse). Mice pretreated with drug solvent but without pseudovirus inoculation served as the healthy control group. Bioluminescence was measured 1 day post infection and visualized in pseudocolor. Finally, mice were sacrificed for experimental analysis immediately after bioluminescence measurement.

#### RNA extraction and real-time PCR

The total RNA was extracted from cultured cells or mouse livers using an RNA prep pure Tissue Kit (Tiangen), and the reverse transcription was performed with RevertAidTM First Strand cDNA Synthesis Kit (Fermentas K1622) according to the manufacturer. The real-time qPCR was then performed on the LightCycler^®^ 96 Real-Time PCR System (Roche) using SYBR Green I Master Mix reagent (Roche) with the primers and using GAPDH as the house-keeping gene. All primer sequences for quantitative PCR assays are listed in Supplementary Table 7.

### Analysis of CTSL activity

The CTSL activities of Huh7 cells infected with control or pseudovirus in the presence of vehicle or different drugs as indicated were measured using a commercially available kit (ab65306) according to the manufacture.

### Statistical analysis

Clinical data were expressed as median (interquartile range (IQR)) or percentage, as appropriate. Comparison of continuous data between groups was determined using Mann–Whitney *U* test. Chi-square (χ^2^) test or Fisher’s exact tests were used for categorical variables as appropriate. To explore the risk factors associated with severity, univariate and multivariate logistic regression models were used. Spearman’s rho test (two-tailed) was used to analyze nonparametric correlations of parameters correlated with SARS-CoV-2 infection and severity of the disease. SPSS for Windows 17.0 and Graphpad prism 7.0 software were used for statistical analysis, with statistical significance set at two-sided *P* < 0.05.

## Supplementary information

Supplementary data R1

## Data Availability

The data that support the findings of this study are available from the corresponding author upon reasonable request.
