# Supplementary material for: Cathepsin L plays a key role in SARS-CoV-2 infection in humans and humanized mice and is a promising target for new drug development
Source: Signal Transduct Target Ther. 2021 Mar 27;6:134. doi: 10.1038/s41392-021-00558-8 (PMC7997800; doi:10.1038/s41392-021-00558-8)
Supplement: Supplementary file 1 — Supplementary data R1 [file 41392_2021_558_MOESM1_ESM.docx]

**Supplementary Materials for**

Cathepsin L plays a key role in SARS-CoV-2 infection in humans and humanized mice and is a promising target for new drug development

Miao-Miao Zhao, Wei-Li Yang, Fang-Yuan Yang, Li Zhang, Wei-Jin Huang, Wei Hou, Chang-Fa Fan, Rong-Hua Jin, Ying-Mei Feng, You-Chun Wang and Jin-Kui Yang

Correspondence to Ying-Mei Feng (yingmeif13@sina.com) or You-Chun Wang (wangyc@nifdc.org.cn) or Jin-Kui Yang ([jkyang@ccmu.edu.cn](mailto:jkyang@ccmu.edu.cn))

Leading contact: Jin-Kui Yang

**This PDF file includes:**

5 figures and 7 tables.


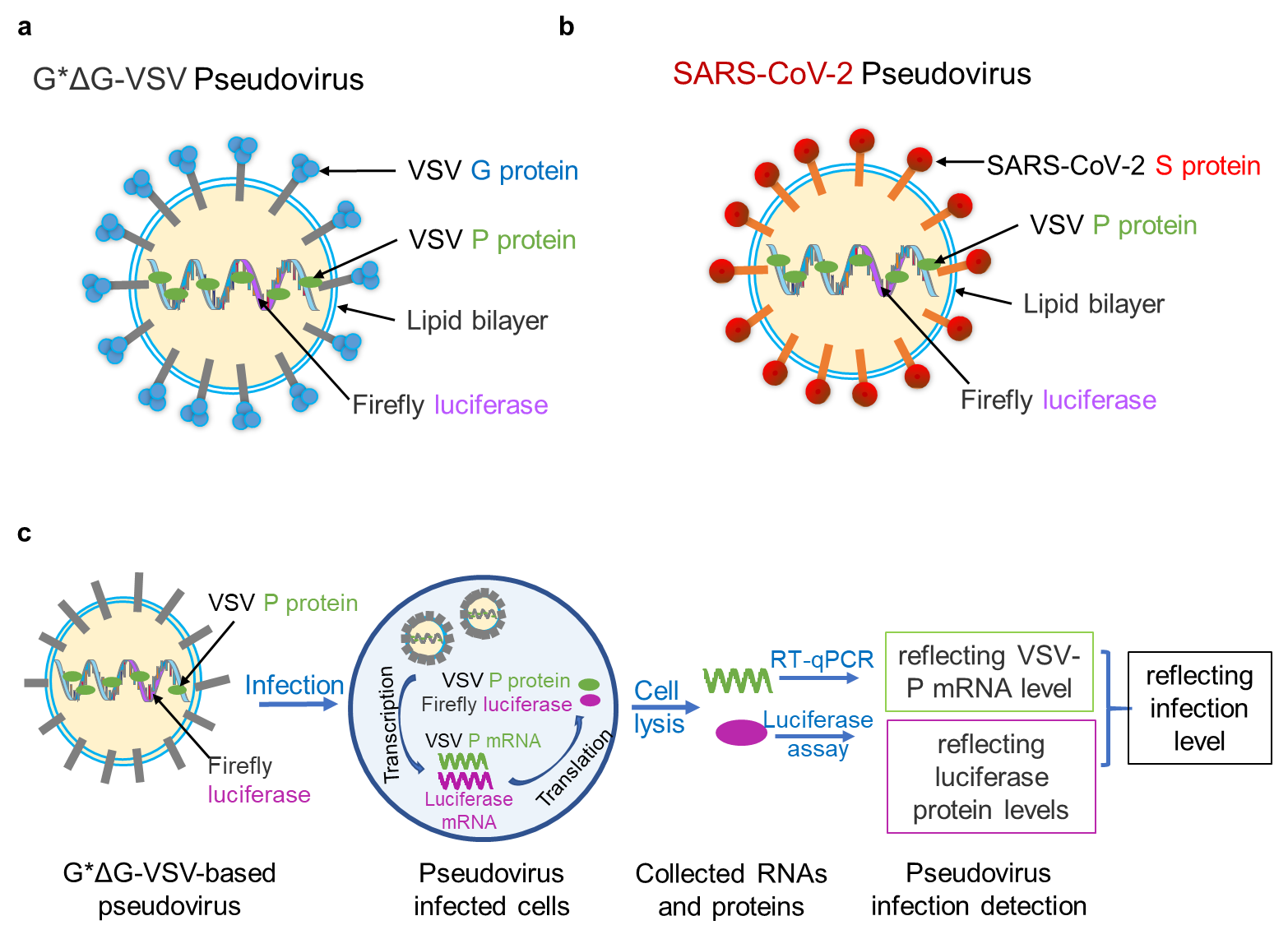


**Supplementary Fig. 1. Schematic diagram of the pseudovirus structure and the pseudovirus infection detection.**

**a,** The G*ΔG-VSV pseudovirus was generate using a recombinant VSV in which the glycoprotein (VSV-G) gene was deleted and replaced with genes encoding firefly luciferase. When VSV-G is expressed transiently in cells infected with these recombinants, VSV pseudotype particles are produced.

**b,** As VSV virions have no specific for selecting the type of membrane protein that can be incorporated into the viral envelope and VSV particles can bud in the absence of G protein. The SARS-CoV-2 pseudovirus was generated by incorporation of the SARS-CoV-2 S protein into the recombinant VSV stated above.

**c,** Schematic of the pseudovirus infection detection. VSV encodes host cell attachment glycoprotein (VSV-G) and phosphoprotein (VSV-P). For the G*ΔG-VSV-based pseudovirus system, the VSV-G gene was deleted and replaced with genes encoding firefly luciferase. The pseudovirus infected cells would therefore express VSV-P and luciferase proteins. Both the luciferase activity and the mRNA level of VSV-P can be used as indicators for pseudovirus infection


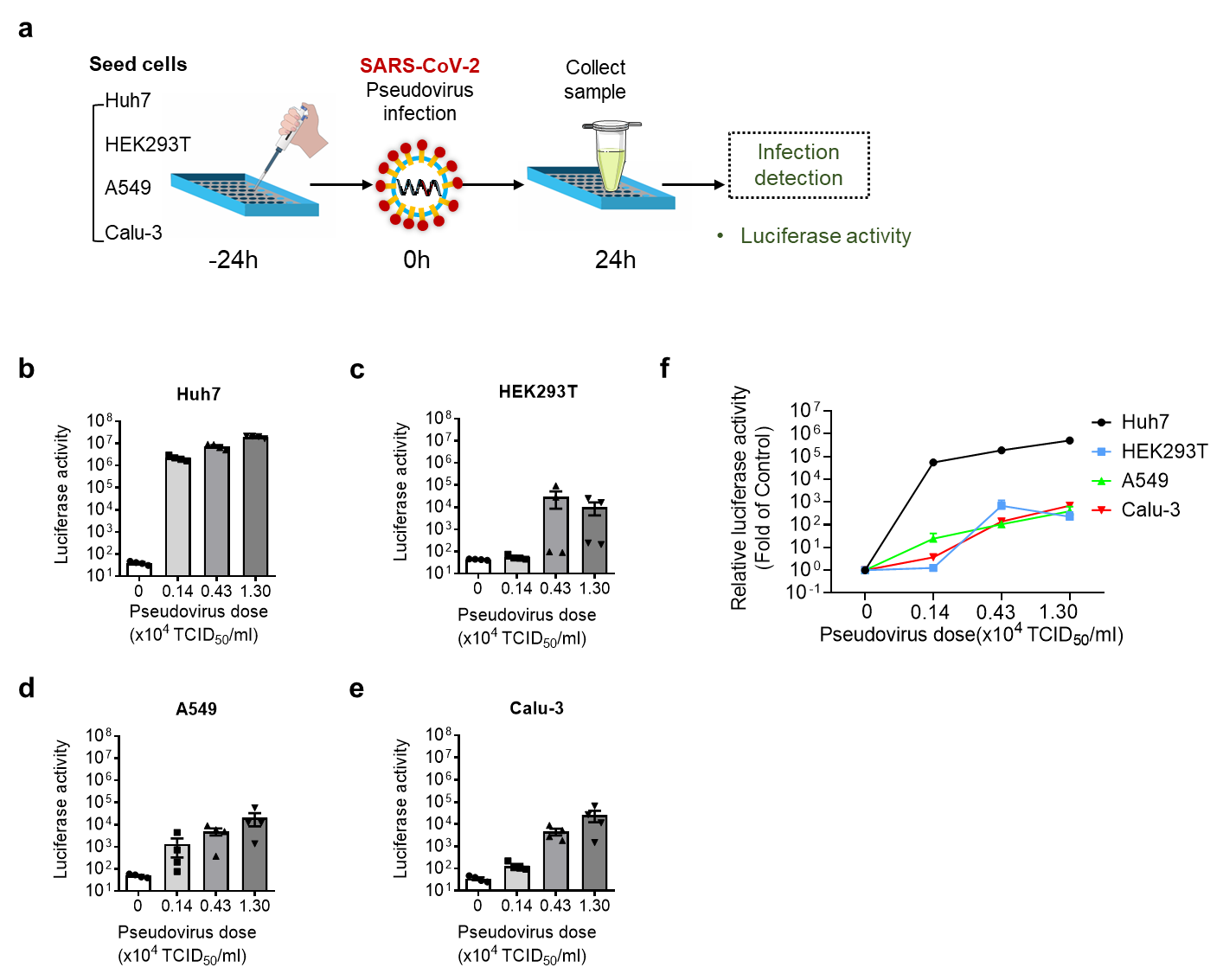


**Supplementary Fig. 2. SARS-CoV-2 pseudovirus infection in different human cell lines.**

**a,** Schematic of the cell line selection assay setup. Huh7, HEK293T, A549 and Calu-3 cells were infected with different doses of SARS-CoV-2 pseudovirus (from 0.14 to 1.30×10^4^ TCID_50_/ml) for 24 h. Cells not infected with pseudovirus were used as control cells.

**b-e,** Pseudovirus infection as evaluated by a luciferase assay and shown as absolute luciferase activity values for Huh7 **(b)**, HEK293T **(c)**, A549 **(d)** and Calu-3 **(e)** cells.

**f,** Pseudovirus infection as indicated by the relative luciferase activity values in Huh7, HEK293T, A549 and Calu-3 cells. Luciferase activity values were normalized to those in the corresponding control cells.

n=4. The data are expressed as the mean ± s.e.m. values.


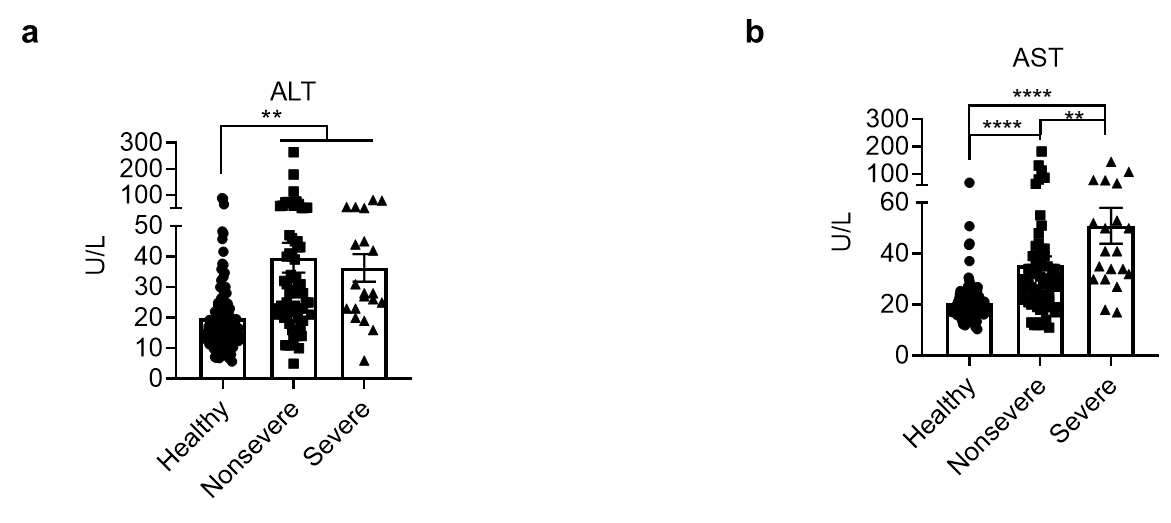


**Supplementary Fig. 3.** **The circulating levels of ALT and AST were increased in COVID-19 patients.**

**a-b,** The ALT and AST levels of healthy volunteers (n=125), nonsevere (n=67) and severe (n=20) patients with COVID-19. Statistical significance was assessed by one-way ANOVA with Tukey's post hoc test for multiple comparisons. ALT, alanine aminotransferase; AST, aspartate aminotransferase.

The data are expressed as the mean ± s.e.m. values. **P*<0.05, ***P* < 0.01, ****P*<0.001, *****P*<0.0001.


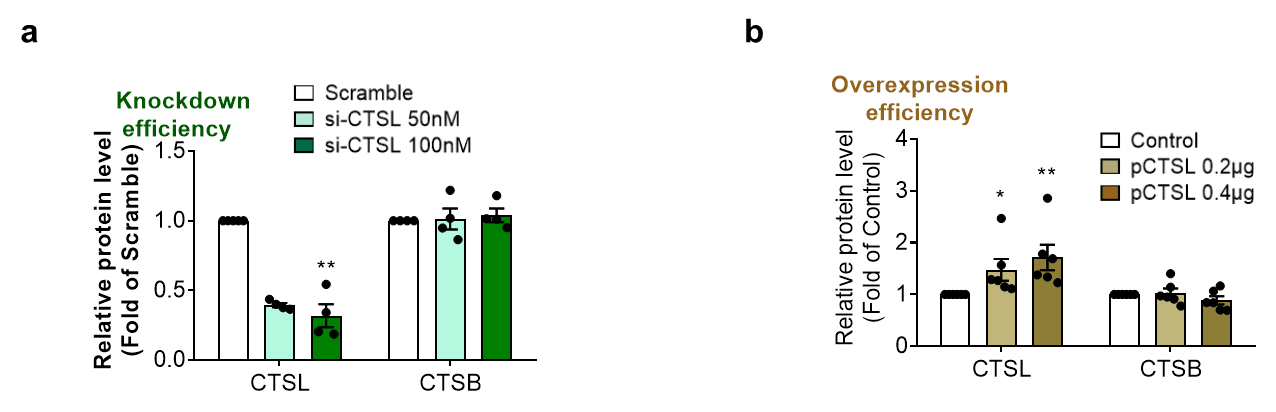


**Supplementary Fig. 4. CTSL knockdown or overexpression efficiencies validation at the protein level in vitro.**

**a,** Dose-dependent knockdown of CTSL by siRNAs without affecting CTSB expression at the protein level by ELISA assay. n=4. Statistical significance was assessed by the Kruskal-Wallis test with Dunn’s post hoc test.

**b,** Dose-dependent overexpression of CTSL with a plasmid encoding the CTSL gene without affecting CTSB expression at the protein level by ELISA assay. n=6. Statistical significance was assessed by the Kruskal-Wallis test with Dunn’s post hoc test.

The data are expressed as the mean ± s.e.m. values. *P<0.05, **P < 0.01.


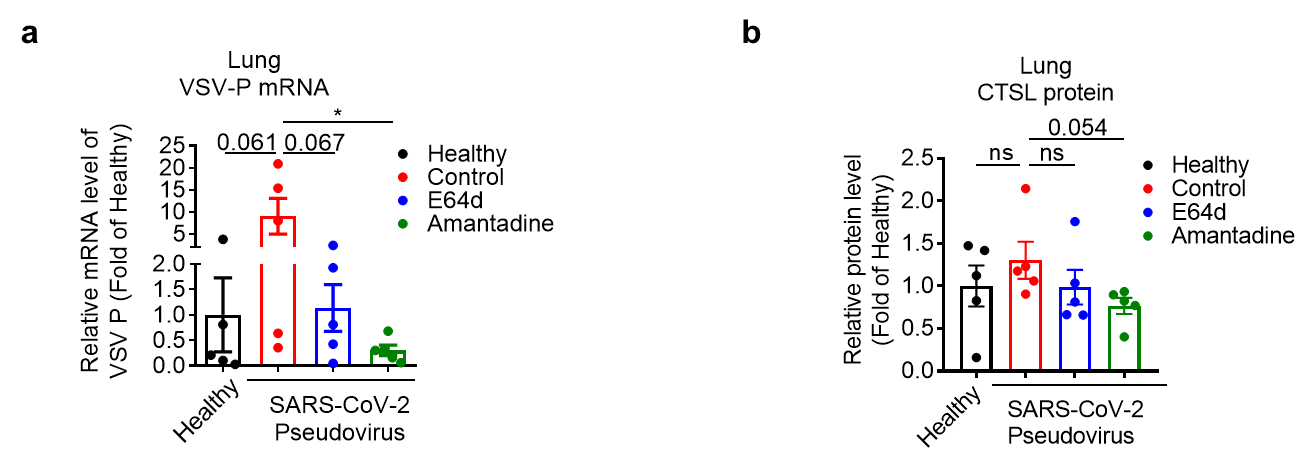


**Supplementary Fig. 5. The lungs were not infected by pseudovirus via tail vein injection in humanized mice.**

**a,** Pseudovirus infection as indicated by the VSV-P mRNA levels in the lungs in each group. Statistical significance was assessed by one-way ANOVA with Tukey's post hoc test for multiple comparisons.

**b**, Pulmonary CTSL protein levels in each group. Statistical significance was assessed by the Kruskal-Wallis test with Dunn’s post hoc test.

n=5. The data are expressed as the mean ± s.e.m. values. **P*<0.05, ***P* < 0.01.

**Supplementary Table 1. Demography, clinical and laboratory parameters of patients with COVID-19.**

|  | **All patients**  **(n=87)** | **Non-severe**  **(n=67)** | **Severe**  **(n=20)** | ***P* value** |
| --- | --- | --- | --- | --- |
| **Age—yrs** | 50 (38-64) | 47 (36-60) | 66 (49-76) | **.000**† |
| **Male sex—n (%)** | 38 (43.7) | 28 (41.8) | 10 (50.0) | .516# |
| **Symptoms** |  |  |  |  |
| **Fever—n (%)** | 64 (73.6) | 50 (74.6) | 14 (70.0) | .681# |
| **Cough—n (%)** | 53 (60.9) | 39 (58.2) | 14 (70.0) | .343# |
| **Sputum—n (%)** | 21 (24.1) | 14 (20.9) | 7 (35.0) | .116! |
| **Dyspnea—n (%)** | 6 (6.9) | 2 (3.0) | 4 (20.0) | **.014**! |
| **Fatigue—n (%)** | 33 (37.9) | 28 (41.8) | 5 (25.0) | .351# |
| **Dysorexia—n (%)** | 10 (11.5) | 6 (9.0) | 4 (20.0) | .111! |
| **Concomitances** |  |  |  |  |
| **Diabetes—n (%)** | 7 (8.0) | 5 (7.5) | 2 (10.0) | .658! |
| **Hypertension—n (%)** | 18 (20.7) | 11 (16.4) | 7 (35.0) | .112! |
| **Routine blood tests** |  |  |  |  |
| **Haemoglobin—g/l** | 138 (125-145) | 139 (128-145) | 133 (121-148) | .366† |
| **Platelet count—×10^9^/l** | 190 (157-233) | 192 (162-233) | 158 (137-233) | .144† |
| **White blood cell count—×10^9^/l** | 4.1 (3.5-5.6) | 4.1 (3.5-5.3) | 5.6 (3.5-7.2) | .142† |
| **Neutrophil percent—%** | 64.3 (51.5-72.1) | 61.7 (50.5-67.9) | 75.7 (66.8-84.1) | .**000**† |
| **Lymphocyte percent—%** | 25.7 (18.8-35.4) | 28.7 (21.7-36.7) | 16.8 (9.4-23.6) | **.000**† |
| **Biochemical parameters** |  |  |  |  |
| **C-reactive protein—mg/l** | 17.4 (3.3-43.5) | 11.3 (2.4-24.7) | 52.7 (33.3-118.1) | **.000**† |
| **Alanine aminotransferase—****U/l** | 28 (21-46) | 28 (21-46) | 28 (23-51) | .634† |
| **Aspartate aminotransferase—U/l** | 30 (22-42) | 28 (22-38) | 41 (30-66) | **.005**† |
| **Albumin—g/l** | 36.7 (33.0-39.8) | 37.7 (34.8-40.0) | 33.0 (29.5-34.4) | **.000**† |
| **Creatine kinase—U/l** | 78 (46-123) | 70 (46-118) | 89 (49-264) | .181† |
| **Creatine kinase–MB—ng/ml** | 0.29 (0.14-0.71) | 0.27 (0.11-0.48) | 0.64 (0.26-1.40) | **.003**† |
| **Myoglobin—ng/ml** | 46.5 (30.3-72.5) | 36.0 (29.5-57.0) | 83.0 (52.0-182.0) | **.000**† |
| **Creatinine—μmol/l** | 64.5 (55.0-77.8) | 64.0 (54.5-75.0) | 66.0 (55.0-88.0) | .366† |

Data are median (IQR) or n (%). P values were calculated by Mann-Whitney U test (†), χ² test (#), or Fisher’s exact test(!), as appropriate for group comparison analyses.

**Supplementary Table 2. Demography and laboratory parameters of heathy volunteers.**

|  | **Healthy**  **(n=125)** |
| --- | --- |
| **Age—yrs** | 49 (38-55) |
| **Male sex—n (%)** | 57 (45.6) |
| **Biochemical parameters** |  |
| **Hemoglobin A1c (%)** | 5.3 (5.1-5.5) |
| **Alanine aminotransferase—U/l** | 16 (12-23) |
| **Aspartate aminotransferase—U/l** | 19 (16-23) |
| **Albumin—g/l** | 45.1 (43.5- 47.4) |
| **Creatine kinase—U/l** | 93 (74-128) |
| **Creatinine—μmol/l** | 68.4 (59.3-78.6) |
| **Total cholesterol (mmol/l)** | 4.84 (4.11-5.45) |
| **HDL cholesterol (mmol/l)** | 1.27 (1.14-1.54) |
| **LDL cholesterol (mmol/l)** | 2.97 (2.38-3.44) |
| **Triglycerides (mmol/l)** | 1.17 (0.81-1.76) |

Data are median (IQR) or n (%).

**Supplementary Table 3. Comparative analysis of the demographic and clinical characteristics of COVID-19 patients and healthy volunteers.**

|  | **Healthy**  **(n=125)** | **Patients**  **(n=87)** | ***P* value** |
| --- | --- | --- | --- |
| **Age—yrs** | 49 (38-55) | 50 (38-64) | 0.102† |
| **Male sex—n (%)** | 57 (45.6) | 38 (43.7) | 0.782# |
| **Biochemical parameters** |  |  |  |
| **Alanine aminotransferase—U/l** | 16 (12-23) | 28 (21-46) | **.000**† |
| **Aspartate aminotransferase—U/l** | 19 (16-23) | 30 (22-42) | **.000**† |
| **Albumin—g/L** | 45.1 (43.5- 47.4) | 36.7 (33.0-39.8) | **.000**† |
| **Creatine kinase—U/l** | 93 (74-128) | 78 (46-123) | **.036**† |
| **Creatinine—μmol/l** | 68.4 (59.3-78.6) | 64.5 (55.0-77.8) | .197† |

Data are median (IQR) or n (%). P values were calculated by Mann-Whitney U test (†) or χ² test (#), as appropriate for group comparison analyses.

**Supplementary Table 4. Circulating parameters correlated with SARS-CoV-2 in patients with COVID-19.**

|  | **All patients**  **(n=87)** | **Non-severe**  **(n=67)** | **Severe**  **(n=20)** | ***P* value** |
| --- | --- | --- | --- | --- |
| **ACE2—ng/ml** | 9.6 (7.8-11.5) | 9.4 (7.4-11.7) | 10.3 (8.5-11.5) | .292† |
| **Ang (1-7) —ng/ml** | 0.54 (0.34-0.81) | 0.58 (0.37-0.91) | 0.45 (0.24-0.56) | **.020**† |
| **CTSL—ng/ml** | 1.71 (1.00-3.44) | 1.44 (0.88-2.55) | 4.03 (1.87-7.83) | **.000**† |
| **CTSB—ng/ml** | 0.52 (0.36-0.73) | 0.56 (0.38-0.74) | 0.39 (0.27-0.58) | **.020**† |

Data are median (IQR). P values were calculated by Mann-Whitney U test (†) for group comparison analyses.

**Supplementary Table 5.** **Nonparametric correlations of parameters correlated with SARS-CoV-2 infection and severity of the disease.**

|  | **Severity** | **CTSL** | **CTSB** | **ACE2** | **Ang (1-7)** | **Age** | **Gender** | **Diabetes** | **HBP** |
| --- | --- | --- | --- | --- | --- | --- | --- | --- | --- |
| **Severity** | 1.00 |  |  |  |  |  |  |  |  |
| **CTSL** | **0.44 (0.000)** | 1.00 |  |  |  |  |  |  |  |
| **CTSB** | **-0.24 (0.030)** | -0.07 (0.564) | 1.00 |  |  |  |  |  |  |
| **ACE2** | -0.11 (0.315) | 0.15 (0.173) | -0.07 (0.543) | 1.00 |  |  |  |  |  |
| **Ang (1-7)** | -0.21 (0.064) | **-0.26 (0.018)** | 0.07 (0.568) | 0.05 (0.655) | 1.00 |  |  |  |  |
| **Age** | **0.37 (0.000)** | **0.62 (0.000)** | **-0.22 (0.045)** | **0.24 (0.029)** | **-0.46 (0.000)** | 1.00 |  |  |  |
| **Gender** | 0.04 (0.718) | 0.02 (0.886) | 0.12 (0.304) | 0.11 (0.351) | 0.02 (0.829) | 0.05 (0.640) | 1.00 |  |  |
| **Diabetes** | 0.05 (0.658) | 0.20 (0.079) | -0.22 (0.053) | -0.02 (0.871) | -0.04 (0.704) | 0.14 (0.205) | -0.01 (0.964) | 1.00 |  |
| **HBP** | 0.14 (0.189) | **0.37 (0.001)** | -0.15 (0.188) | 0.06 (0.620) | -0.21 (0.065) | **0.46 (0.000)** | 0.01 (0.942) | **0.37 (0.000)** | 1.00 |

Data are correlation coefficient (p value). Spearman's rho test (2-tailed)

**Supplementary Table 6. siRNA sequence mixture against human CTSL.**

| **Dulex Name** | **Sense Seq (5’-3’)** | **Anti Seq (5’-3’)** |
| --- | --- | --- |
| **CTSL-1 (human)** | AGGCGAUGCACAACAGAUUAUTT | AUAAUCUGUUGUGCAUCGCCUTT |
| **CTSL-2 (human)** | CCAAAGACCGGAGAAACCAUUTT | AAUGGUUUCUCCGGUCUUUGGTT |
| **CTSL-3 (human)** | GUGGGAGAAGAACAUGAAGAUTT | AUCUUCAUGUUCUUCUCCCACTT |
| **CTSL-4 (human)** | AGGAGAAGGCCCUGAUGAATT | UUCAUCAGGGCCUUCUCCUTT |
| **CTSL-5 (human)** | GGAUUAUGCUUUCCAGUAUTT | AUACUGGAAAGCAUAAUCCTT |
| **CTSL-6 (human)** | GGGCAUGGGUGGCUACGUATT | UACGUAGCCACCCAUGCCCTT |

**Supplementary Table 7. List of oligonucleotide primer pairs used in real time RT-PCR analysis.**

| **primer** | **species** | **Forward (5’-3’)** | **Reverse (5’-3’)** |
| --- | --- | --- | --- |
| **CTSL** | human | AAACTGGGAGGCTTATCTCACT | GCATAATCCATTAGGCCACCAT |
| **CTSB** | human | ACAACGTGGACATGAGCTACT | TCGGTAAACATAACTCTCTGGGG |
| **VSV-P** | VSV | TCTCGTCTGGATCAGGCGG | TGCTCTTCCACTCCATCCTCTTGG |
| **GAPDH** | human | GAAGGTGAAGGTCGGAGT | CATGGGTGGAATCATATTGGAA |
| **GAPDH** | mouse | CTGAGTATGTCGTGGAGTCTAC | GTTGGTGGTGCAGGATGCATTG |
